# Use of a Ceramic Membrane to Improve the Performance of Two-Separate-Phase Biocatalytic Membrane Reactor

**DOI:** 10.3390/molecules21030345

**Published:** 2016-03-14

**Authors:** Giuseppe Ranieri, Rosalinda Mazzei, Zhentao Wu, Kang Li, Lidietta Giorno

**Affiliations:** 1 Institute on Membrane Technology, CNR-ITM, C/o University of Calabria, Via P. Bucci 17/C, 87036 Rende (CS), Italy; g.ranieri@itm.cnr.it (G.R.); r.mazzei@itm.cnr.it (R.M.); 2 Department of Chemical Engineering, Imperial College London, South Kensington Campus, SW7 2AZ London, UK; zhentao.wu@imperial.ac.uk (Z.W.); kang.li@imperial.ac.uk (K.L.)

**Keywords:** biocatalytic membrane, immobilized lipase, ceramic biocatalytic membrane, two-separate-phase biocatalytic membrane reactor

## Abstract

Biocatalytic membrane reactors (BMR) combining reaction and separation within the same unit have many advantages over conventional reactor designs. Ceramic membranes are an attractive alternative to polymeric membranes in membrane biotechnology due to their high chemical, thermal and mechanical resistance. Another important use is their potential application in a biphasic membrane system, where support solvent resistance is highly needed. In this work, the preparation of asymmetric ceramic hollow fibre membranes and their use in a two-separate-phase biocatalytic membrane reactor will be described. The asymmetric ceramic hollow fibre membranes were prepared using a combined phase inversion and sintering technique. The prepared fibres were then used as support for lipase covalent immobilization in order to develop a two-separate-phase biocatalytic membrane reactor. A functionalization method was proposed in order to increase the density of the reactive hydroxyl groups on the surface of ceramic membranes, which were then amino-activated and treated with a crosslinker. The performance and the stability of the immobilized lipase were investigated as a function of the amount of the immobilized biocatalytst. Results showed that it is possible to immobilize lipase on a ceramic membrane without altering its catalytic performance (initial residual specific activity 93%), which remains constant after 6 reaction cycles.

## 1. Introduction

In the last decade inorganic membranes have attracted considerable interest in the field of membrane technology. The excellent properties of inorganic membranes in terms of chemical, mechanical and thermal resistance make them suitable materials to develop innovative devices, promoting new research from production to their application. The versatility of these membranes has allowed their use in various applications such as hydrogen production and separation [1,2], propane dehydrogenation [3], filtration for corrosive fluids [4] and for the development of high temperature reactors [5], membrane contactors [6], biosensors [7] and biocatalytic membrane reactors [8,9].

The present study explored the suitability of inorganic hollow fibers to improve the performance of a biocatalytic membrane reactor (BMR), often developed by using polymeric membranes mainly due to their lower costs. On the other hand, inorganic membranes are more resistant to organic solvents [10] and therefore they might be more suitable for biocatalytic membranes operating in a biphasic system. Recent works [11,12] reported very good performance from polymeric and hybrid membranes with regard to organic solvents, even though some problems still remain to be solved such as polymer plasticisation, swelling and clustering [12]. For BMR development a highly uniform and stable nanostructured porous matrix is needed; in fact the non-homogeneity of the membrane does not permit a good enzyme distribution during chemical immobilization or maintain a constant residence time during the biocatalytic reaction. For this reason we used ceramic membranes to develop the BMR.

Phase inversion has been widely adopted for the production of polymeric hollow fibre membranes [13], and has been further developed to fabricate ceramic counterparts after modifying the suspension compositions and incorporating an additional sintering process Asymmetric hollow fiber configuration offers a high surface area/volume ratio, after counting in the surface area of the self-organised micro-channels, which promotes an increased loading of the enzyme and a higher oil/water interfacial surface preferred in a biphasic system. Many studies have been carried out on BMRs by employing different kinds of polymeric membranes and biocatalysts [14], with limited studies available on BMRs using inorganic support, summarized in Table 1.

One of the most studied biocatalysts is lipase, which is active in different reactions such as hydrolysis, esterification, and transesterification, with high enantioselective properties [22]. Lipase is an interfacial molecule with phase transfer catalytic properties, meaning it performs to the best of its abilities in an oil/water interface [23]. Besides, most lipase substrates have low water solubility, so the reaction is conventionally performed in a multiphase membrane reactor where the membrane acts not only as a support for the enzyme, but also as a separation-and-contacting unit between the two immiscible phases. This configuration permits the continuous removal of the selected product, via extraction, from the reaction microenvironment, thus promoting reaction yield to a higher level. The need to use organic solvents to perform the reaction of immobilized enzyme could be a problem for polymeric membrane reactors in a long term operation. Prolonged contact time with an organic solvent might swell the polymeric material, reducing the performance of the biocatalytic reactor in terms of stability. The use of inorganic membranes in a multiphase membrane reactor may overcome these limitations. Furthermore, inorganic membranes are easy to regenerate and reuse for subsequent enzyme immobilization, after long term use and enzyme deactivation. So far, there is insufficient information about the catalytic performance of lipase immobilized in inorganic membranes.

In the present work, a biocatalytic membrane reactor was developed by immobilizing lipase from *Candida rugosa* on “home-made” alumina hollow fiber membranes through covalent binding. Alumina hollow fibre membranes were prepared according to the method described by Benjamin *et al.* [13]. The fabricating method was modified in order to obtain an asymmetric structure with finger-like supporting layers functioning as high throughput microreactors in series. Lipase from *Candida rugosa* was immobilized using (3-aminopropyl) triethoxysilane (APTES)—glutaraldehyde (GA) modification approach. Since the effect of APTES concentration on the immobilization process has already been studied by Miletić *et al.* [24], in this study the silanization process was performed with a constant concentration of APTES, addressing the effects of hydroxylation and GA concentration on enzyme immobilization. The influence of immobilized lipase amount on the specific activity and biocatalytic reactor performance was also studied. The catalytic activity was evaluated in a two-separate-phase biocatalytic membrane reactor (TSP-BMR).

## 2. Results and Discussion

### 2.1. Alumina Hollow Fiber Membrane Preparation

**Figure 1:**
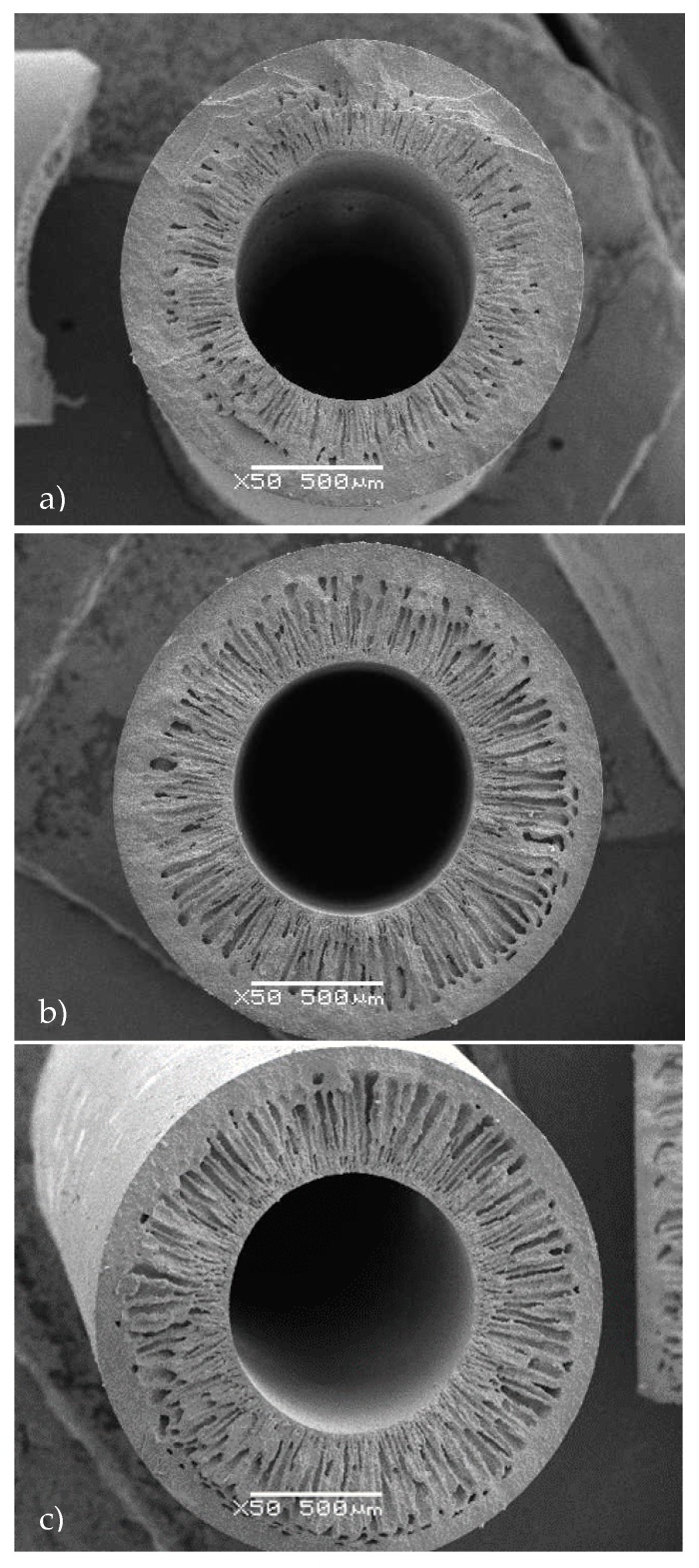
Cross section of hollow fiber membranes resulting from an internal coagulant flow rate of 5 mL/min (**a**); 10 mL/min (**b**) and 20 mL/min (**c**).

In this section, the results of the fabrication and characterization of alumina hollow fibre membranes are presented. Membranes were prepared using various internal coagulant flow rates, which affected the length of the finger-like voids as well as the membrane porosity. Figure 1 shows morphologies of asymmetric alumina hollow fiber membranes resulting from an internal coagulant flow rate of 5, 10, 20 mL/min. As shown, finger-like voids extend from the lumen surface across approximately 50% (Figure 1a), 80% (Figure 1b) and 90% (Figure 1c) of the fiber cross-section.

The fibers showed uniform wall thickness along the fiber length. The outer diameter (OD) and internal diameter (ID) of the fibers were 1830 μm (±0.08) and 920 μm (±0.01), respectively. The hollow fibers possess a unique asymmetric structure composed of long finger-like voids and a thin sponge-like layer. The formation of finger-like voids at the lumen surface is initiated by instabilities at the interface between the non-solvent and the suspension. Finger-like void growth then proceeds as a result of non-solvent influx into the suspension, similar to the viscous fingering phenomenon. Because of the high concentration of non-solvent at the interface, polymer precipitation instantaneous results in a rapid, large increase in the viscosity of the suspension in this region. Consequently, the suspension viscosity exceeds the critical value at which further morphological change may take place and the size of the entrances to the finger-like voids is determined at this point. Non-solvent influx through these entrances into the suspension results in finger-like void growth. However, the polymer precipitation rate within the suspension is lower than that at the interface with non-solvent due to the less availability of non-solvent in this region. As a result, the suspension viscosity remains below the critical value, thus longer finger-like void growth may proceed, giving rise to the characteristic finger-like shape. Finger-like void growth cannot proceed above a critical suspension viscosity and consequently growth is halted when this value is exceeded. Exposure of the outer fiber surface to the atmosphere can cause an increase in viscosity in this region due to simultaneous solvent evaporation and non-solvent condensation from the surrounding atmosphere. For fibers prepared in this work, the critical suspension viscosity is exceeded in the outer region of the fiber before immersion in non-solvent takes place. Consequently, finger-like void growth is not observed at the outer fiber surface and a sponge-like structure is formed. SEM images clearly confirm the progressive increase in the thickness of the finger-like region as the internal coagulant flow rate increases. Accordingly, ultrapure water permeability increases with the same trend from 319 (±19) L/hm^2^bar to 1136 (±186) L/hm^2^bar and an intermediate value of 507(±51) L/hm^2^bar. The membranes with intermediate permeability (Figure 1b) were selected for the development of the biocatalytic membrane reactor due to the high surface area/volume ratio for enzyme loading as well as intermediate water affinity in order to permit better contact of the membrane with the organic solvent in the biphasic system. The void volume of biocatalytic membrane reactors are summarized in Table 2.

### 2.2. Membrane Functionalization and Enzyme Immobilization

Prior to enzyme immobilization, as explained in Materials and Methods section, blank experiments were carried out on native membranes to evaluate the exclusive enzyme covalent binding. No adsorbed or entrapped enzyme was detected.

**Figure 2:**
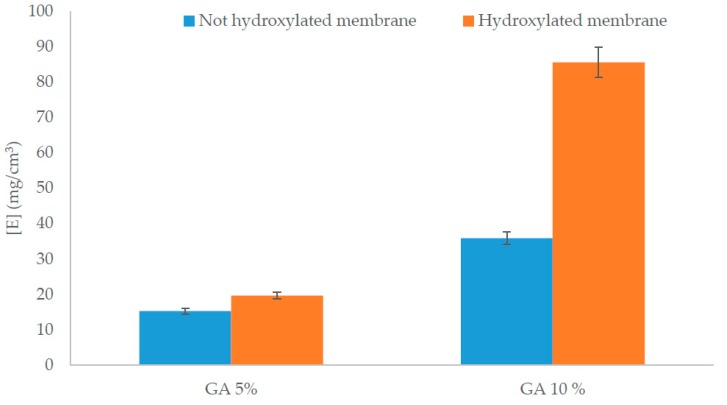
Immobilized enzyme amount depending on hydroxylation and GA concentration.

In addition, on the same native membranes, no color variation of ninhydrin solution was observed, which confirms that there is no interference between the matrix and ninhydrin assay. Performing the assay on Al-APTES, a variation of ninhydrin solution from yellow to purple was detected. As expected, a colorless solution was obtained by carrying out the assay on Al-APTES-GA, because no more exposed amino-groups on the membrane surface are presents. The amount of the amino groups per unit surface generated after the functionalization process was 1.26 × 10^−3^ (±1.56 × 10^−4^) mmol/cm^2^. The effect of the functionalization on water permeability was investigated. Compared to the initial permeability value of native membranes, a permeability decreased by about 37% (±1%) after the silanization process and by about 47% (±3%) after the treatment with glutaraldehyde. Afterwards, lipase immobilization was carried out as described in the Materials and Methods section. In this last case, a further permeability reduction of about 88% (±3%) was detected, confirming the presence of the covalently bonded enzyme on the membrane. The results achieved, in terms of immobilized enzyme amount, by performing hydroxylation process on membrane and by varying GA concentration are shown in Figure 2.

Immobilized enzyme concentration increases to about 58% by increasing the GA concentration when the fibers are non-hydroxylated. The situation is different in the case of hydroxylated fibers where the amount of immobilized enzyme increases to about 80.7%. By comparing non-hydroxylated fibers and the hydroxylated ones at the same GA concentration, it may notice a slight increase of 14.6% of immobilized enzyme amount by using 5% GA concentration. On the other hand, a significant increase of about 60.5% of immobilized amount occurs between non-hydroxylated fibers and hydroxylated fibers by performing the covalent immobilization with 10% GA concentration. During hydroxylation process the density of hydroxyl groups on the membrane should increase, consequently more silanol molecules should react. Therefore, more amino-groups should be exposed on the hydroxylated fibers in contrast to the non-hydroxylated ones. Probably, by using 5% GA concentration, amino-groups formed on the surface are not completely quenched. A further functionalization of amino-groups may occur by using 10% GA concentration with a final higher amount of immobilized enzyme. An additional effect that could cause higher enzyme immobilization is given by the formation of GA multilayer on membrane due to the GA polymerization [25,26]. This additional layer can cause enzyme immobilization by covalent attachment on available aldehyde groups, as well as retention by unspecific interactions as observed in the literature in the case of highly activated glutaraldehyde supports [27].

### 2.3. Lipase Activity Measurements

**Figure 3:**
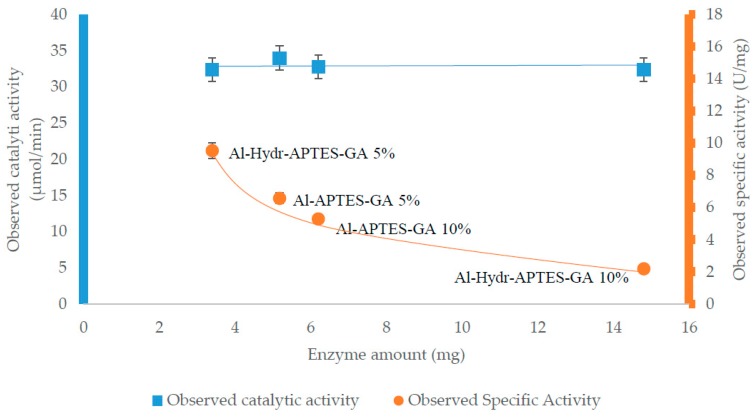
Trend of observed specific activity depending on immobilized enzyme amount.

Performance of free and immobilized lipase was tested in a multiphase stirred tank reactor (STR) and in a two-separate-phase biocatalytic membrane reactor respectively. The observed specific activity of free lipase was 10.2 (±1) U/mg. Observed specific activity of immobilized lipase was evaluated in order to compare the performance with the free one. Specific activities obtained from biocatalytic membrane reactors with different immobilized enzyme amounts were also investigated. Figure 3 presents the behavior of the observed catalytic activity and observed specific activity as a function of immobilized enzyme amount. Immobilization strategy is also highlighted.

**Figure 4:**
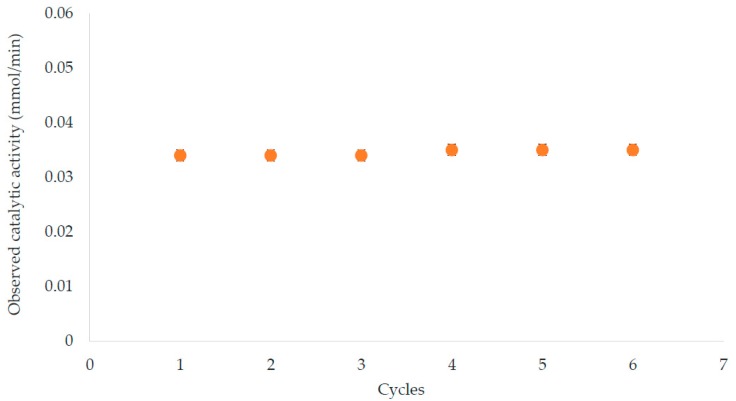
Observed catalytic activity of immobilized lipase after different reaction cycles.

Results show that catalytic activity is constant whilst specific activity decreases with increase of enzyme amount. This means that the same amount of immobilized lipase works at the interface and the enzyme immobilized in excess negatively affects the observed specific activity. Both crowding phenomena and mass transport properties contribute to the overall observed effect. Indeed, the best results in terms of observed specific activity, were achieved in the case of the lower amount of immobilized lipase, *i.e.*, in the case of Al-Hydr-APTES-GA 5% where the enzyme retains an observed specific activity of about 93% compared to the free one (10.2 ± 1 U/mg). Therefore, a lower enzyme amount was not investigated since the observed specific activity (9.5 ± 0.5 U/mg) was similar to the one of the free enzyme. In the case of Al-Hydr-APTES-GA 10%, the highest immobilized enzyme amount causes a considerably decay of observed specific activity while Al-APTES-GA 5% and Al-APTES-GA 10% retains an observed specific activity of about 65% and 52%, respectively. Besides, the observed specific activity of the immobilized lipase does not undergo significant variations after 6 cycles of measurements, for a total time of about 18 days (Figure 4). After each reaction cycle any permeability change was observed.

The behaviour confirms that the amount of immobilized biocatalyst is a key parameter to be optimized in biocatalytic membrane reactor development since higher amount of enzyme does not implies better performance of the reactor system. By comparing results obtained in this work with a previous study about lipase from *Candida rugosa*, where the TSP-BMR was developed by immobilizing the enzyme onto polymeric membranes through physical entrapment [28], it is possible to confirm that better performance of the immobilized lipase was detected. In that case, about 6.7 mg of lipase from *Candida rugosa* was immobilized onto asymmetric polyamide hollow fiber membranes and the reaction was performed recirculating 200 mL of organic phase (olive oil) with an axial flow rate of 80 mL·min^−1^ along the shell side and 600 mL of aqueous phase (50 mM phosphate buffer pH 8.00) with an axial flow rate of 400 mL/min along the lumen side. Volumetric reaction rate of free and immobilized lipase from *Candida rugosa* resulting from the two studies are reported in Table 3.

Native lipase from *Candida rugosa* previously studied in a STR from Giorno *et al.* [28] has a lower activity respect from the native lipase examined in the present study, probably due to lower purity of the enzymatic powder. Independently from a lower volumetric reaction rate, a decrease of about 99% occurs after the immobilization of lipase onto polymeric membranes by physical entrapment, as reported in Table 4. On the other hand, volumetric reaction rate of lipase from *Candida rugosa* studied in this work decreases only about 34% after covalent immobilization onto alumina membranes. Despite of the covalent attachment of the enzyme on the inorganic support, which in most cases causes a decrease of the enzymatic performance due to modifications of the native conformation of protein, as reported in literature [14], the enzyme preserves better performance than in the polyamide membrane where it was immobilized by physical entrapment. The suitability of inorganic membranes for TSP-BMR development was also demonstrated after enzyme removal by cleaning procedure. In fact, the initial membrane permeability (507 ± 51 L/hm^2^bar) was completely restored (527 ± 68.5 L/hm^2^bar). Therefore, this procedure allows the reuse of the fibers for enzyme immobilization in case of biomolecule denaturation during industrial processes.

## 3. Materials and Methods

### 3.1. Materials and Chemicals

Aluminium oxide powders 1 μm (alpha, 99.9% metal basis, surface area 6–8 m^2^/g), 0.3 μm (gamma–alpha, 99.9% metal basis, surface area 15 m^2^/g), 0.05 μm (gamma–alpha, 99.5% metal basis, surface area 32–40 m^2^/g) and 0.01 μm (gamma–alpha, 99.98% metal basis, surface area 100 m^2^/g) were purchased from Alfa Aesar (a Johnson Matthey company, London, UK) and were used as supplied. Polyethersulfone (Radal A300, Ameco Performance, Houston, FL, USA), *N*-methyl-2-pyrrolidone (HPLC grade, Rathbone, London, UK) and Arlacel P135 (Polyethyleneglycol 30-dipolyhydroxystearate, Uniqema, Paterson, NJ, USA) were used as binder, solvent and additive, respectively. Tap water and de-ionized water were used as the external and internal coagulants, respectively. Hydrogen peroxide solution (30% wt from Sigma-Aldrich, Milan, Italy) and Sulfuric acid 96% (purchased from VWR, UK) were used for the preparation of piranha solution. Absolute Ethanol (purchased from VWR), (3-Aminopropyl) triethoxysilane 99% (APTES) and Glutaraldehyde solution (both purchased from Sigma-Aldrich) were used for the functionalization process of the alumina membrane. Ninhydrin and Glycine (purchased from Sigma-Aldrich) were used respectively as reagent and standard for the characterization of functionalized membranes. Sodium dihydrogen phosphate anhydrous (NaH_2_PO_4_) and disodium hydrogen phosphate anhydrous (Na_2_HPO_4_) (purchased from Sigma-Aldrich) were used to prepare phosphate buffer solution at pH 7.0. Lipase from *Candida rugosa* (E.C.3.1.1.3, 65 kDa, Sigma-Aldrich) was used for covalent immobilization process. Since lipase solution was not pure, a preliminary centrifugation at 3000 rpm for 15 min of the initial protein solution (after two h mixing of the protein solution) was made and the supernatant recovered was used. BCA protein assay (Sigma-Aldrich) was used to evaluate protein solution concentration. NaOH and NaOCl (purchased from Carlo Erba, Milan, Italy) were used for the preparation of the cleaning solutions.

### 3.2. Equipment

#### 3.2.1. Alumina Hollow Fiber Membranes Preparation

Two stainless steel syringes (Harvard 200 mL) are placed on the top of the system where the spinning suspension and internal coagulant were loaded respectively. The extrusion rate of the spinning suspension and the internal coagulant were accurately controlled and monitored by two individual Harvard PHD 22/2000 Hpsi syringe pumps, ensuring the uniformity of the prepared precursor fibers. Spinning suspension is forced to pass through a tube-in-orifice spinneret (ID 1.2 mm, OD 3.0 mm) connected to the syringes and a 120 L coagulation bath collects the native fiber precursors. A CARBOLITE furnace was used for the calcination of the fibers precursors.

#### 3.2.2. Surface Activation and Enzymatic Covalent Immobilization

The equipment used to perform the surface activation and the enzymatic immobilization is constituted by hollow fiber membranes assembled in a Pyrex module 100 mm long. In addition to the module, the system is constituted by a peristaltic pump (P) to feed the reagent solution to the lumen side of the membranes, pressure gauges (PG) to measure inlet and outlet pressure and a beaker to collect retentate and permeate solution. Same system configuration was used to perform the membrane cleaning procedure.

#### 3.2.3. Stirred Tank Reactor (STR) Setup

The reaction took place in a tank with 21 mL of total reaction mixture containing a mixture which consists in 19 mL of phosphate buffer 50 mM, 1 mL of olive oil and 1mL of enzyme solution 0.8 g/L. The mixture was stirred at 300 rpm by a magnetic stirrer to disperse the two immiscible phases and create an oil-water reaction interface. The reaction tank was kept at 30 °C in a thermostatic bath. The reaction was monitored by measuring the extracted fatty acids into the aqueous phase produced during tryglycerides hydrolysis. The specific activity is expressed as U/mg enzyme. One unit (U) is defined as 1 μmol of fatty acid produced per minute. A Mettler DL25 automatic titrator was used to measure the fatty acids extracted into the aqueous phase titrating with NaOH (20 mM). The specific activity was calculated as the slope in the straight line of the micromoles of reaction products *versus* time and then normalized by the mass of enzyme.

#### 3.2.4. Two-Separate-Phase Biocatalytic Membrane Reactor (TSP-BMR)

**Figure 5:**
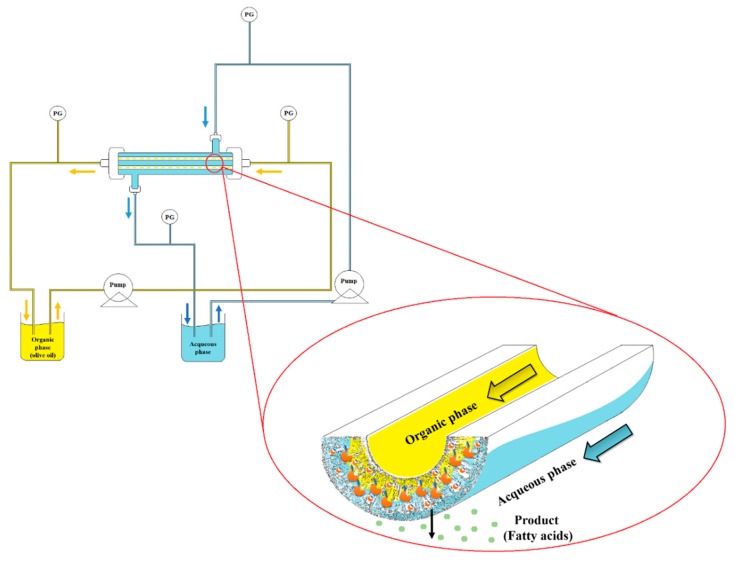
Schematic representation of two-separate-phase biocatalytic membrane reactor (TSP-BMR).

The two-separate-phase biocatalytic membrane reactor is depicted in Figure 5. It is constituted by a module containing two membranes and it is equipped with suitable connections for shell and lumen circuits. Gear pumps were used to supply the organic and aqueous phases to the reactor. Two control panels, with valves, flow meters and pressure gauges, were used to control, separately, the shell and lumen circuits. The system was kept at 30 °C by submerging the module and the tanks containing the two phases in thermostatic baths. 250 mL of extra-virgin olive oil (natural substrate for the lipase) was used as organic phase, while 1 L of phosphate buffer pH 7 was used as aqueous phase.

### 3.3. Operation Mode

#### 3.3.1. Preparation of Alumina Hollow Fiber Membranes

Alumina hollow fiber membranes were prepared by a combined phase inversion and sintering technique [13]. Briefly, dispersant (Arlacel P135 1.3 wt %) was dissolved in NMP/water solutions and then aluminum oxide powders (58.7 wt %) at a ratio of 1:2:7 (0.01 μm:0.05 μm:1 μm) were added to the mixture. The dispersion was rolled/milled with 20 mm agate milling balls for 48 h. Milling was continued for a further 48 h after the addition of polyetheresulfone (6.1 wt %). The suspension was then transferred to a gas tight reservoir and degassed under vacuum until no bubbles could be seen at the surface. After degassing, the suspension was transferred to the stainless steel syringe and was extruded through a tube-in-orifice spinneret into the coagulation bath containing water (a non-solvent for the polymer) with an air-gap of 15 cm. Deionized water was used as the internal coagulant and 10 mL/min was used as the extrusion flow rate. The extrusion rate of the spinning suspension was 7 mL/min. The fiber precursors were left in the external coagulation bath overnight to allow for completion of phase inversion. They were then immersed in an excess of tap water which was replaced periodically over a period of 48 h in order to remove traces of NMP. Finally, the fiber precursors were calcined in air to yield ceramic hollow fiber membranes. The temperature was increased from room temperature to 600 °C at a rate of 2 °C/min and held for 2 h, then to the target temperature of 1350 °C at a rate of 5 °C/min and held for 4 h. The temperature was then reduced to room temperature at a rate of 3 °C/min. Table 4 summarizes the parameters used for membrane preparation.

#### 3.3.2. Membrane Functionalization and Enzyme Immobilization

Hydroxylated and non-hydroxylated membranes were used. APTES treatment were carried out in order to activate the surface with silanol group. Afterwards, cross-linking binding (Glutaraldehyde) was performed by using two different concentrations of reagent (5% and 10% *v*/*v*). Finally enzyme was immobilized on hydroxylated and non-hydroxylated fibers as well as after cross-linker binding. Prior to perform silanization, alumina hollow fiber membranes were immersed in freshly prepared piranha solution (30% H_2_O_2_ and 96% H_2_SO_4_ 1:3 *v*/*v*) for 15 min in order to clean the fiber and to increase density of hydroxyle groups on the membrane surface, as reported by Nesrine Aissaoui *et al.* [18]. Membranes were then thoroughly washed in ultrapure (UP) water and dried under nitrogen gas flow. After hydroxylation process, membrane fibers were assembled in a glass module, as reported in Figure 5. In order to activate the surface with silanol groups, both hydroxylated alumina fibers (Hydr-Al) and non-hydroxylated ones (Al) were reacted with a fresh ethanol solution of APTES (5% APTES, 5% water and 90% pure ethanol) for 2 h at room temperature [7] with an axial velocity of 0.033 m/s and a transmembrane pressure (TMP) of 0.05 bar. Fibers were then rinsed several times with deionized water. Silanized fibers were then treated with two Glutaraldehyde solution (5% and 10% *v*/*v*) for 2 h at room temperature with an axial velocity of 0.033 m/s and a transmembrane pressure of 0.05 bar. Fibers were then rinsed several times with deionized water in the same conditions of axial velocity and pressure. Covalent binding occurs through formation of a Schiff base between aldehydic terminal group of glutaraldehyde and amino group of silane. Non-hydroxylated alumina fibers functionalized with APTES and 5% or 10% GA will be refered in the text as *Al-APTES-GA5%* and *Al-APTES-GA10%*, respectively.

**Figure 6:**
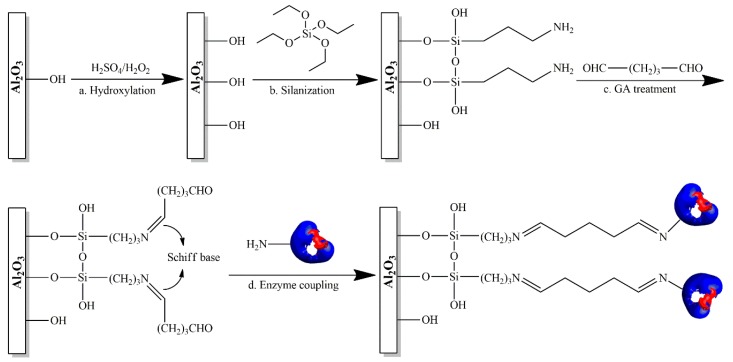
Stages involved during immobilization process: (**a**) Hydroxylation process by immersing inorganic hollow fiber membranes in a piranha solution; (**b**) Silanization process by treating membranes with APTES solution; (**c**) Cross-linker binding between glutaraldheyde and amino-group of silane by forming Schiff base; (**d**) Enzyme covalent binding on the inorganic hollow fiber membranes.

*Al-Hydr-APTES-GA5%* and *Al-Hydr-APTES-GA10%* will be used respectively to refer the hydroxylated fibers functionalized with APTES and 5% or 10% GA. The chemical functionalized membranes were then used for enzyme immobilization. Phosphate buffer pH 7 was used to prepare the enzymatic solution. The protein concentration was 0.8 g/L as estimated by BCA protein assay. Functionalized membranes were used to perform covalent immobilization. Enzymatic solution was fed to the membrane, at room temperature, along the lumen circuit with an axial velocity of 0.033 m/s and a transmembrane pressure of 0.05 bar for 16 h. The enzyme solution was forced to pass through the membrane porous wall. Membranes were then rinsed with phosphate buffer four times in order to remove any trace of the enzyme, until any absorbance was measured at 280 nm. In order to confirm that enzymatic immobilization is only due to the covalent immobilization, blank experiments were carried out to be sure that any interaction between enzyme and native membrane materials was occurring. This process was carried out by ultrafiltering enzyme solution through native membrane, applying a pressure of 0.2 bar. Each step of the immobilization process is illustrated in Figure 6.

In order to evaluate the quantity of immobilized lipase on the membrane, the protein concentration in the initial, final, and washing solution was measured by BCA test kit (Sigma). The amount of immobilized protein was determined by mass balance according to the following equation:

C*i*V*i* = C*_f_* V*_f_* + C*_ws_*V*_ws_* + m (1)

where m indicates the immobilized protein mass in the membrane, C and V represent the concentration and volume, respectively; the subscripts *i*, *f* and *ws* indicate the initial, final, and washing solutions, respectively. The immobilized mass was then normalized by the membrane void volume to estimate the immobilized enzyme amount related to membrane volume. Membrane void volume represents the biocatalytic membrane reactor volume. It was calculated as the volume of evaporated water from calibrated pieces of wet membrane by thermal balance.

#### 3.3.3. Two-separate-phase Biocatalytic Membrane Reactor (TSP-BMR)

After the enzyme was immobilized within the membrane, the lumen circuit was recirculated with olive oil while aqueous phase was recirculated along the shell side, both with an axial flow rate of 300 mL/min. The organic phase pressure was maintained at 0.6 bar and the aqueous phase pressure was maintained at 0.35 bar; therefore, a transmembrane pressure of 0.25 bar was applied from the organic phase to the aqueous phase, to prevent the aqueous phase passing through the membrane into the organic phase. The system was kept at constant temperature (30 ± 1 °C) by submerging the module and the tanks containing the two phases in thermostatic baths. The membrane acts as a catalytic interface, separating the two phases which remained in contact within the membrane pores. The enzyme reaction took place within the membrane phase and the product fatty acids formed by the reaction tryglicerides hydrolysis are extracted in the aqueous phase by diffusion. The reaction was monitored by titration of the produced fatty acids extracted in the aqueous phase with NaOH (20 mM) as previously reported for the batch system. The performance of the membrane reactor was expressed reported as “observed specific activity” on the basis of fatty acids present in the bulk aqueous phase, measured in terms of units expressed as U/mg enzyme. One unit is defined as 1 μmol of fatty acid produced per minute. The fatty acids present in the bulk depend on both reaction and transport properties. Therefore, the observed specific activity approaches the intrinsic specific activity when mass transfer is negligible and the system works in reaction limited regime.

#### 3.3.4. Membrane Cleaning Procedure

Thanks the high chemical and temperature resistance of the inorganic membranes, it was possible to apply a cleaning procedure by using a strong basic detergent in order to regenerate native properties of the membranes. Membranes were cleaned using a detergent solution obtained with 20 wt % of NaOH, 2.5 wt % of NaOCl and 77.5 wt % of water. 2% (*v*/*v*) of this prepared solution was pressed through the membrane at a TMP of 0.4 bar and at 80 °C for 1 h. Membranes were then rinsed several time with deionized water until the pH of the washing waters was unaltered. Ultrapure water permeability tests were used to monitor the recovered native properties.

### 3.4. Characterizations

#### 3.4.1. Characterization of Prepared Membranes

SEM characterization was conducted for sintered fibers which were flexed at ambient temperature until a fracture occurred prior to being mounted on an SEM slide. Samples were gold coated under vacuum for 3 min at 20 mA (EMITECH Model K550) and SEM images at varying magnifications were collected (JEOL JSM-5610 LV). Water permeability of the fibers was determined by ultrafiltration of 15 MΩ pure water at different TMP.

#### 3.4.2. Characterization of Grafted Alumina Membranes Surface

A rapid and sensitive method for the qualitative and quantitative determination of free amino groups created on the alumina surface has been developed. The method developed is a modification of Kaiser’s method [27,29,30]. The technique involves the reaction of the amine with ninhydrin; under carefully controlled conditions Ruhemann’s purple dye anion is formed.

Briefly, small pieces of Al-Hydr-APTES and Al-APTES membranes (3.5 cm) were introduced in the tubes together with 1 mL of the ninhydrin solution and absolute ethanol up to a total volume of 6 mL. The mixtures were heated at 100 °C for 5 min and then cooled down in a cool water bath [31]. Blank experiments were performed also by using Al-fibers in order to check that no interference occurs with the membrane matrix and to confirm that the purple color is only due to the presence of amino groups. Ninhydrin tests were subsequently repeated on Al-APTES-GA fibers in order to confirm that the cross-linker binding occurred by covering the free amino groups on the surface by producing a colorless solution. Different solutions of known concentration of glycine were used to establish a slope of the linear curve between absorbance and concentration necessary for the quantification measurement of the amino groups. In order to study the effect of the immobilization strategy on the membrane behaviour, UP water permeability was evaluated after treatment with GA and finally after the enzyme was covalently immobilized.

## 4. Conclusions

In this study, the suitability of alumina hollow fiber membranes to improve the performance of a two-separate-phase biocatalytic membrane reactor was demonstrated. An appropriated method in order to immobilize lipase from *Candida rugosa* by covalent binding on alumina membrane was identified. Results confirmed that a higher immobilized enzyme amount does not correspond to a higher observed specific activity, probably due to a crowding phenomenon. The best results, in terms of observed specific activity, were obtained by performing the immobilization process through hydroxylation of alumina membranes followed by a 5% glutaraldehyde treatment (Al-Hydr-APTES-GA 5%) in which immobilized lipase retains about 93% of the observed specific activity. In the mentioned conditions it is possible to carry out olive oil hydrolysis without any significant variation of enzymatic specific activity for about 6 reaction cycles with a running period of about 18 days.

## Figures and Tables

**Table 1:** Examples of BMRs using inorganic support.

Immobilized Protein	Catalyzed Reaction	Inorganic Material Support	Ref.
Candida antarctica lipase B	Hydrolysis of p-nitrophenyl palmitate	Alumina	[9]
Rat hepatic microsomal CYP2E1	Para-nitrophenol hydroxylation	Alumina	[15]
Alliinase	Conversion of alliin in alliicin	Alumina	[16]
Glucose-6-phosphate dehydrogenase	Oxidation of Glucose-6 phosphate (G6P)	Alumina	[17]
		Silica	[18]
Laccase	Oxidation of 2,2′-azino-bis-(3 ethylbenzothiazoline-6-sulfonic acid)	Titania	[19]
Urease	Urea hydroxylation	Alumina	[20]
Glutathione transferase	Glutathione conjugation to 1-chloro-2,4-dinitrobenzene	Alumina	[21]

**Table 2:** Virgin membrane void volumes of the biocatalytic membrane reactors.

Biocatalytic Membrane Reactor	Membrane Void Volume (cm^3^)
Al-APTES-GA 5%	0.34 (±0.06)
Al-APTES-GA 10%	0.17 (±0.03)
Al-Hydr-APTES-GA 5%	0.19 (±0.03)
Al-Hydr-APTES-GA 10%	0.16 (±0.02)

**Table 3:** Volumetric reaction rate resulting from the study about lipase immobilized with different approaches on different matrices.

Biocatalytic Membrane Reactors	Volumetric Reaction Rate (mmol/dm^3^h)	
	Free Lipase	Immobilized Lipase
In a previous work with Polyamide membranes [28]	6.95 (±0.61)	0.044 (±0.008)
In the present work with Alumina membranes	12.4 (±0.62)	7.2 (±0.36)

**Table 4:** Operative conditions used for membranes preparation.

Sample No	Internal Coagulant Flow Rate	Polymer:Powder Ratio	Air Gap	Spinning Suspension Flow Rate	Sintering Temperature
**1**	5 mL·min^−1^	1:10	150 mm	7 mL·min^−1^	1350 °C
**2**	10 mL·min^−1^				
**3**	20 mL·min^−1^				

## Abbreviations

The following abbreviations are used in this manuscript:

APTES: (3-Aminopropyl) triethoxysilane
GA: Glutaraldehyde
TSP-BMR: Two-separate-phase biocatalityc membrane reactor
Al-APTES-GA 5%: Non-hydroxylated alumina fibers functionalized with APTES and GA 5%
Al-APTES-GA 10%: Non-hydroxylated alumina fibers functionalized with APTES and GA 10%
Al-Hydr-APTES-GA 5%: Hydroxylated alumina fibers functionalized with APTES and GA 5%
Al-Hydr-APTES-GA 10%: Hydroxylated alumina fibers functionalized with APTES and GA 10%
STR: Stirred tank reactor
